# Limited Add-On Effects of Unilateral and Bilateral Transcranial Direct Current Stimulation on Visuo-Motor Grip Force Tracking Task Training Outcome in Chronic Stroke. A Randomized Controlled Trial

**DOI:** 10.3389/fneur.2021.736075

**Published:** 2021-11-11

**Authors:** Benedikt Taud, Robert Lindenberg, Robert Darkow, Jasmin Wevers, Dorothee Höfflin, Ulrike Grittner, Marcus Meinzer, Agnes Flöel

**Affiliations:** ^1^Neurocure Cluster of Excellence, Charité University Medicine, Berlin, Germany; ^2^Department of History, Philosophy and Ethics of Medicine, Heinrich Heine University, Düsseldorf, Germany; ^3^Berlin Institute of Health at Charité, Charité University Medicine, Berlin, Germany; ^4^Institute of Biometry and Clinical Epidemiology, Charité University Medicine, Berlin, Germany; ^5^Department of Neurology, University Medicine Greifswald, Greifswald, Germany; ^6^German Centre for Neurodegenerative Diseases, Site Greifswald/Rostock, Greifswald, Germany; ^7^Center for Stroke Research, Charité University Medicine, Berlin, Germany

**Keywords:** chronic stroke, tDCS, motor function, motor rehabilitation, hand function

## Abstract

**Background:** This randomized controlled trial investigated if uni- and bihemispheric transcranial direct current stimulation (tDCS) of the motor cortex can enhance the effects of visuo-motor grip force tracking task training and transfer to clinical assessments of upper extremity motor function.

**Methods:** In a randomized, double-blind, sham-controlled trial, 40 chronic stroke patients underwent 5 days of visuo-motor grip force tracking task training of the paretic hand with either unilateral or bilateral (*N* = 15/group) or placebo tDCS (*N* = 10). Immediate and long-term (3 months) effects on training outcome and motor recovery (Upper Extremity Fugl-Meyer, UE-FM, Wolf Motor Function Test, and WMFT) were investigated.

**Results:** Trained task performance significantly improved independently of tDCS in a curvilinear fashion. In the anodal stimulation group UE-FM scores were higher than in the sham group at day 5 (adjusted mean difference: 2.6, 95%CI: 0.6–4.5, *p* = 0.010) and at 3 months follow up (adjusted mean difference: 2.8, 95%CI: 0.8–4.7, *p* = 0.006). Neither training alone, nor the combination of training and tDCS improved WMFT performance.

**Conclusions:** Visuo-motor grip force tracking task training can facilitate recovery of upper extremity function. Only minimal add-on effects of anodal but not dual tDCS were observed.

**Clinical Trial Registration:** https://clinicaltrials.gov/ct2/results?recrs=&cond=&term=NCT01969097&cntry=&state=&city=&dist=, identifier: NCT01969097, retrospectively registered on 25/10/2013.

## Background

Stroke is the leading cause of acquired disability worldwide (1–3). The most common consequence of a stroke is a hemiparesis of the contralesional upper limb. Approximately 80% of all stroke patients are initially affected and persistent (chronic) impairment of upper extremity functioning is observed in ~40% of stroke patients, resulting in reduced quality of life, participation in societal activities, and lower odds for successful vocational reintegration (4). Numerous motor rehabilitation strategies, like physical or occupational therapy, have been investigated that aim at improving upper extremity function [for reviews see (5, 6)]. However, treatment effects can be highly variable and frequently only small to moderate effect sizes and limited transfer to activities of daily living have been reported (7–10).

Consequently, development of adjunct approaches that aim at enhancing the effects of behavioral interventions, such as non-invasive brain stimulation, have recently received substantial attention. In particular, the combination of behavioral treatment with repetitive transcranial magnetic stimulation (rTMS) or transcranial direct current stimulation (tDCS) can have positive effects on motor rehabilitation after stroke (11, 12). During rTMS, a strong magnetic field is used to induce electrical activity in the underlying brain area. Depending on the frequency, intensity, and duration of the stimulation, rTMS is able to increase or decrease cortical excitability (13). Low frequency stimulation (<1 Hz) results in decreased cortical excitability, whereas high frequency stimulation (<1 Hz) results in increased cortical excitability (13–15). TDCS involves administration of a weak electrical current via scalp attached electrodes. Acute stimulation effects are mediated by modulation of the neural resting-membrane potential, resulting in changes in cortical excitability in underlying cortical regions. Depending on the polarity of the current, tDCS can be used to increase or decrease cortical excitability (16), with anodal tDCS yielding a relative depolarization and cathodal tDCS yielding hyperpolarization of neuronal membranes (17). Long-term effects of the stimulation are thought to depend on synaptic mechanisms mimicking long-term potentiation, which are critical for neural plasticity and learning (18). The current study utilized tDCS because it can be administered concurrently with motor training, while offering a superior safety profile and placebo stimulation condition compared to rTMS (19).

In motor rehabilitation after stroke, three different tDCS electrode set-ups have frequently been used and are based on different assumptions regarding neural recovery (20): (1) Unihemispheric excitatory (“anodal”) tDCS targeting the ipsilesional motor cortex is used to facilitate neural function regions that are spared by the stroke. (2) Unihemispheric inhibitory (“cathodal”) tDCS targeting the contralesional motor cortex is used to reduce activity in regions that may potentially interfere with motor behavior. (3) Bihemispheric “dual” tDCS combines both approaches and aims at facilitating ipsilesional neural activity, while inhibiting contralesional regions (21–25). Several studies have reported beneficial tDCS effects on motor rehabilitation using anodal (26–29), cathodal (30–32), or dual set-ups (21, 22, 33). However, few studies have directly compared the effects of different set-ups (31, 34, 35). Moreover, with regard to upper extremity functioning, substantial previous research has focused on training of whole arm movements (21, 22, 36, 37). However, severely impaired patients may not be able to execute extensor muscle movements which are required for these training paradigms [e.g., constraint-induced movement therapy, CIMT, (38)]. To address this problem, the effectiveness of treatment approaches targeting fine motor control of the paretic hand have been explored (38, 39). Indeed, many severely affected patients are still able to generate whole hand grip force (40, 41) and improvement of grip force is associated with improvements of upper limb functional status (42). Moreover, a previous study demonstrated improvement on the Upper-Extremity Fugl-Meyer (UE-FM) elbow scale through visuo-motor training. However, no additional gains were induced by anodal tDCS (38), possibly related to the low current strength (0.5 mA) that was used. In addition, it has not yet been investigated whether dual tDCS might also have a positive effect on visuo-motor grip force tracking task training.

Therefore, the present study aimed to address three open questions: (1) Does visuo-motor grip force tracking task training improve performance in the trained task, and/or generalizes to clinical assessments of upper extremity function in chronic stroke? (2) If immediate and long-term training gains occur, are they enhanced by concurrent unilateral or bilateral motor cortex tDCS? (3) Does unilateral or bilateral tDCS result in differential transfer effects? We hypothesized that both anodal and dual tDCS would enhance motor training outcome and transfer to clinical assessments of upper motor function. Effects of contralesional cathodal tDCS alone were not examined because negative effects were reported in chronic patients with moderate or severe impairment (43).

## Materials and Methods

Forty patients with chronic (>6 months post-stroke; see Table 1 for details) right or left hemispheric ischemic or hemorrhagic stroke participated in a randomized, double-blind, sham-controlled study. Inclusion criteria were the occurrence of an ischemic or hemorrhagic stroke at least 6 month prior to enrollment; no subsequent strokes; no additional neurological, medical, or psychiatric disorders or contraindication for tDCS (e.g., skull fractures or metal implants); no concurrent use of CNS-affecting drugs and the ability to complete the motor training.

**Table 1:** Demographic information and baseline motor performance.

**Group**	**Age, years**	**Time post-stroke, months**	**Sex (♂/♀)**	**Baseline UE-FM**	**Baseline WMFT**	**Affected hemisphere (right/left)**
Dual	58.3 ± 12.8	21.9 ± 17.2	11/4	47.1 ± 17.9	0.7 ± 0.6	8/7
Anodal	60.3 ± 10.3	28.8 ± 35.3	12/3	46.9 ± 15.0	0.6 ± 0.6	8/7
Sham	60.6 ± 12.9	28 ± 25.1	8/2	43.6 ± 20.7	0.8 ± 0.7	6/4
Mean SMD	0.13	0.19	0.11	0.13	0.17	0.09

*Values are reported as mean ± standard deviation, SMD, standardized mean difference*.

**Figure 1:**
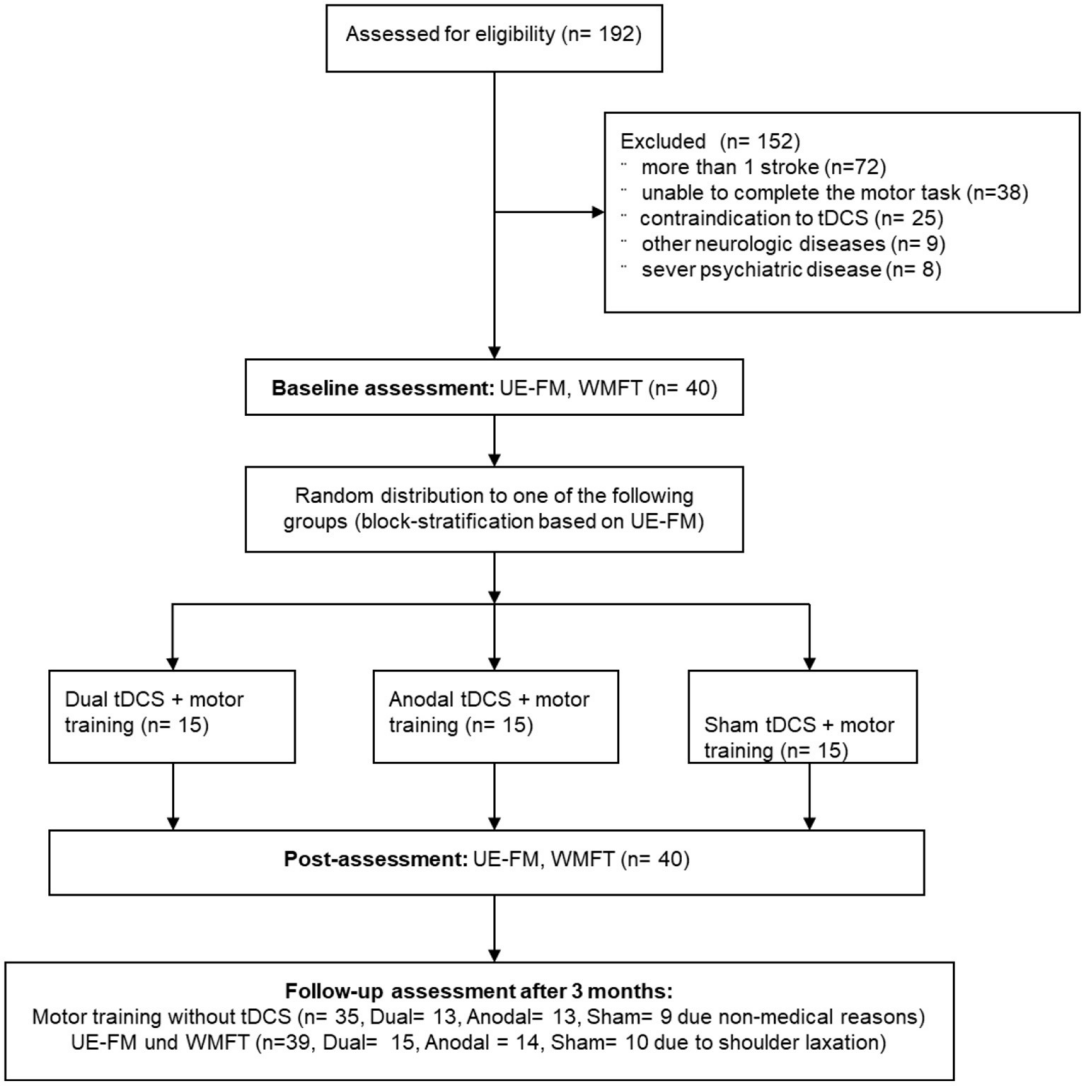
Study flow chart. UE-FM, Upper Extremity Fugl-Meyer; WMFT, Wolf Motor Function Test; tDCS, transcranial direct current stimulation.

Eligible patients were stratified to the stimulation groups by baseline UE-FM score to receive 5 consecutive days of visuo-motor grip force tracking task training with either anodal (*N* = 15), dual (*N* = 15), or placebo (“sham”; *N* = 10) tDCS. Patients, care providers and investigators were blinded to the stimulation conditions. Sample size estimations were based on previous uni- and bihemispheric tDCS studies (22, 24). The study was approved by the local ethics committee. Participants gave written informed consent prior to study inclusion. The trial was registered (NCT01969097). Figure 1 displays the flow-chart of the study.

### Primary and Secondary Research Question

The primary research question was whether unilateral or bilateral tDCS in combination with a visuo-motor grip force tracking task training improves UE-FM scores in stroke patients. Changes in training performance and WMFT scores were secondary outcomes.

### Clinical Assessment

All patients underwent standardized assessments of motor function and impairment using UE-FM and WMFT that were administered prior to and immediately after the intervention. The UE-FM examines multi-joint movements of the upper limb [max. score = 66, lower scores = greater impairment; (44)]. The WMFT comprises 15 time-based items ranging from whole arm movements to fine finger control. WMFT completion times were logarithmized to account for skewed data distribution (45). This score has a maximum value of 2.08 s[log] with lower values reflecting better arm function. All tests (including the training task without-tDCS) were repeated 3 months later to investigate potential long-term effects of tDCS on motor function. Assessments were videotaped and analyzed by two independent raters.

One participant (anodal-group) was excluded from UE-FM/WMFT follow-up assessments due to a shoulder subluxation. Follow-up motor task data of five participants (2 dual, 2 anodal, 1 sham) was not recorded correctly due to technical difficulties and could not be used in the analysis.

### Motor Training

**Figure 2:**
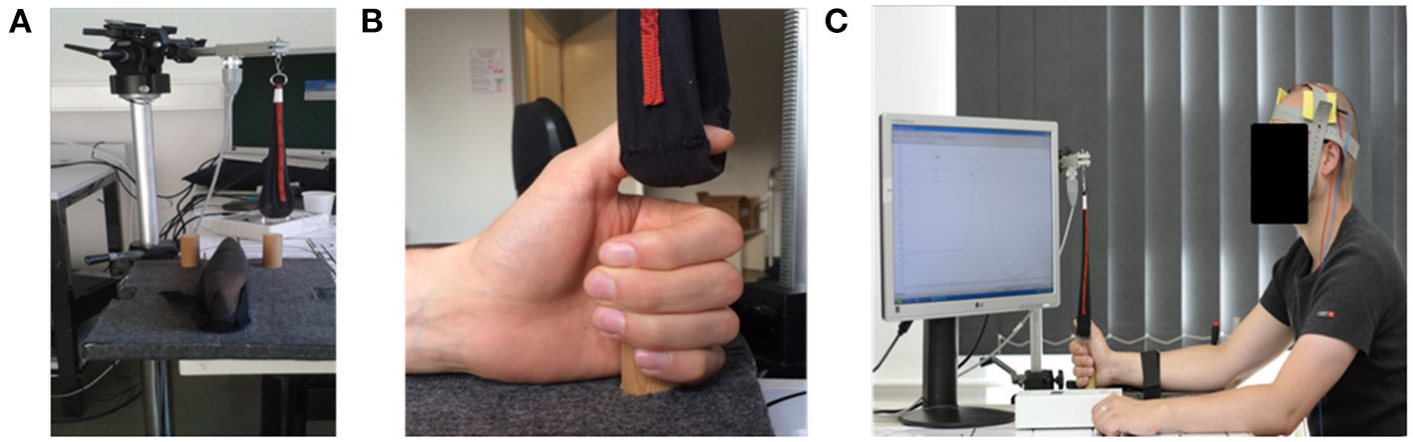
Experimental set-up. **(A)** The Grass® Force Displacement Transducer FT10, the sling to measure the applied strength and the grip board. **(B)** Shows a left hand grasping the wooden grip while the thumb is placed in the sling connected to the Force Displacement Transducer. **(C)** Illustrates the overall set-up with the dual tDCS montage.

Details of the visuo-motor grip force tracking task have been described previously (46). In short, patients performed isometric adductions with their paretic thumb. They sat comfortably in front of a computer screen and were asked to hold on to a wooden grip protruding vertically from the table with their paretic hand. Their thumb was placed in a sling attached to a Grass® Force Displacement Transducer FT10 (Grass Instruments). Velcro straps were used to fixate the forearm in order to minimize unwanted movement. The set-up is displayed in Figure 2. Signal software (Cambridge Electronic Design Ltd.) was used for data acquisition and task presentation. Force displacement was amplified and digitized using a CPT22 AC/DC Straining Gage Amplifier (Grass® Technologies) with an amplification of 2,000 Hz and a high filter of 3 Hz.

At the beginning of each session, the patients abducted their thumb five times as hard as possible in order to establish a maximum force output for each individual session. A target force window was then defined as the range between 30 and 40% of the individual maximum force output and displayed as three horizontal lines (30, 35, and 40%) on the computer screen. Each experimental display was scaled individually based on the patient's individual maximum force output. A horizontal line representing 40% was located 9 cm from the top of the screen, and a line representing 30% was located 13 cm from the top of the screen. In between, a line representing 35% was displayed. The zero force line was positioned at the bottom of the screen.

**Figure 3:**
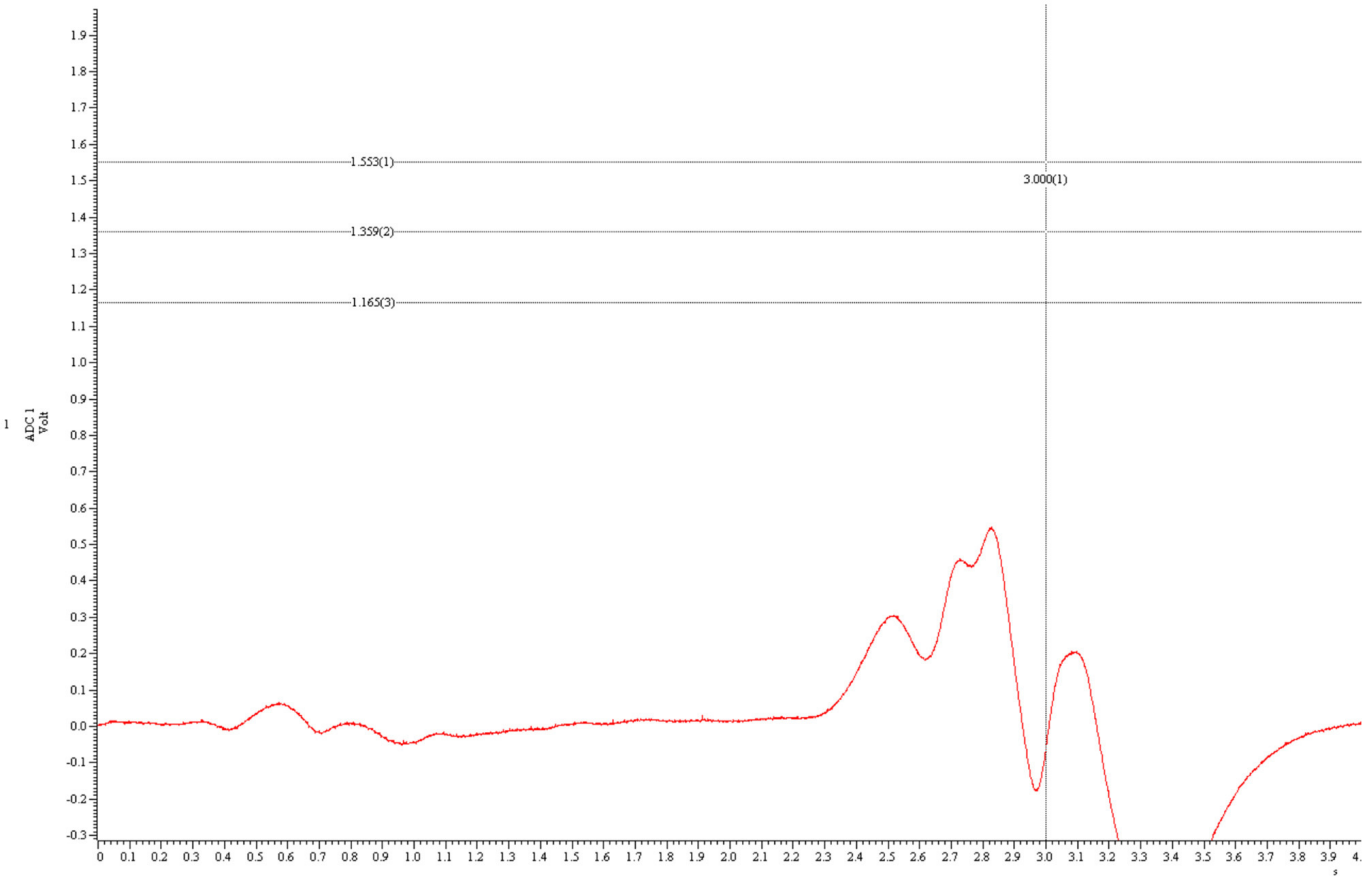
A typical trial run. This figure shows a typical trial where the participant did not apply enough force. The y-axis represents force, the x-axis time. The three horizontal lines represent, from top to bottom, 40, 35, and 30% of the individual maximum force output. The red curved line represents the applied force by the participant. The highest point of the red line lies beneath 30%. Hence, this is a “miss.”

Each trial lasted 4 s and at the 3 s mark a vertical line was displayed as reference for the participants. During each trial, a red line moved in real time across the screen from left to right at zero force. Whenever pressure was applied via the force transducer, the line moved upwards accordingly and returned to zero force after the pressure was released. Participants were asked to abruptly apply pressure via their thumb in a manner that caused the red line to reach its highest point as close as possible to the intersection of the 35% line and a vertical line displayed at the 3 s mark, before returning to zero force. A trial was scored as “hit” if participants managed to place the red line's maximum between the 30% (bottom) and 40% (top) lines. All other trials were scored as “miss.” A typical trial-run is displayed in Figure 3. Since participants were required to apply only 30–40% of their maximum force output, this was considered a visuo-motor grip force tracking task training.

During each motor training session, participants completed 240 trials across eight blocks of 30 trials each. In between blocks they were given a 60 s rest period, resulting in a total training time of ~23 min.

### Direct Current Stimulation

A DC-Stimulator PLUS (NeuroConn, Germany) was used for stimulation with the active anode (5 × 7 cm^2^) placed over the ipsilesional primary motor cortex according to the international 10–20 EEG system (i.e., C3 or C4, depending on site of the lesioned hemisphere). In the unilateral (anodal tDCS only) condition, the cathode (10 × 10 cm^2^) was placed over the contralesional supraorbital ridge. The large size of the cathode rendered the stimulation functionally inert during this condition. In the dual condition, a smaller active cathode (5 × 7 cm^2^) was placed over the contralesional primary motor cortex. In the sham condition, the electrode set-up was pseudo-randomly assigned to participants (either anodal or dual) and balanced across the group. Direct current was increased to 1 mA over 10 s and lasted for 23 min in the anodal and dual conditions. In the sham condition, it was ramped down after 30 s. This procedure has resulted in successful participant blinding in previous studies [see (47)]. The tDCS parameters were within established safety guidelines (48). A second investigator configured the DC-Stimulator PLUS in order to ensure investigator blinding.

### Statistical Analysis

SPSS 22 (IBM Corp. Released 2013) and R [packages: lme4, r2glmm, tidyverse, ggeffects (49–53)] and a two-sided significance level (*alpha* = 0.05) was employed. Separate linear mixed models (54) investigated effects of the active stimulation conditions compared to sham tDCS on performance (motor task, UE-FM, WMFT). Time points (motor task: training-days_1−5_, follow-up; UE-FM/WMFT: baseline, post, follow-up) were level-one units nested in different individuals (level-two units). Random intercept models tested differences between the stimulation conditions. A squared centered time variable (TIME^2^) tested for curvilinear learning effects in the regression model for motor task. The Time × Stimulation interaction assessed whether the slopes differed between groups. Baseline UE-FM scores and training blocks were covariates in the motor task analysis. For models testing stimulation effects on UE-FM and WMFT, the respective baseline values were included as covariates as well as the time point of measurement (day 5, 3 month follow up) and the interaction of stimulation group and time point. In order to investigate effects of active stimulation, we also conducted linear mixed models testing the combined stimulation effect of anodal and dual stimulation vs. sham tDCS (this analysis was reported in the Supplementary Material along with training outcomes for individual patients, see Supplementary Figures 1–3). There was no adjustment for multiple testing and *p*-values. See Supplementary Materials for full model estimates.

## Results

### UE-FM

**Figure 4:**
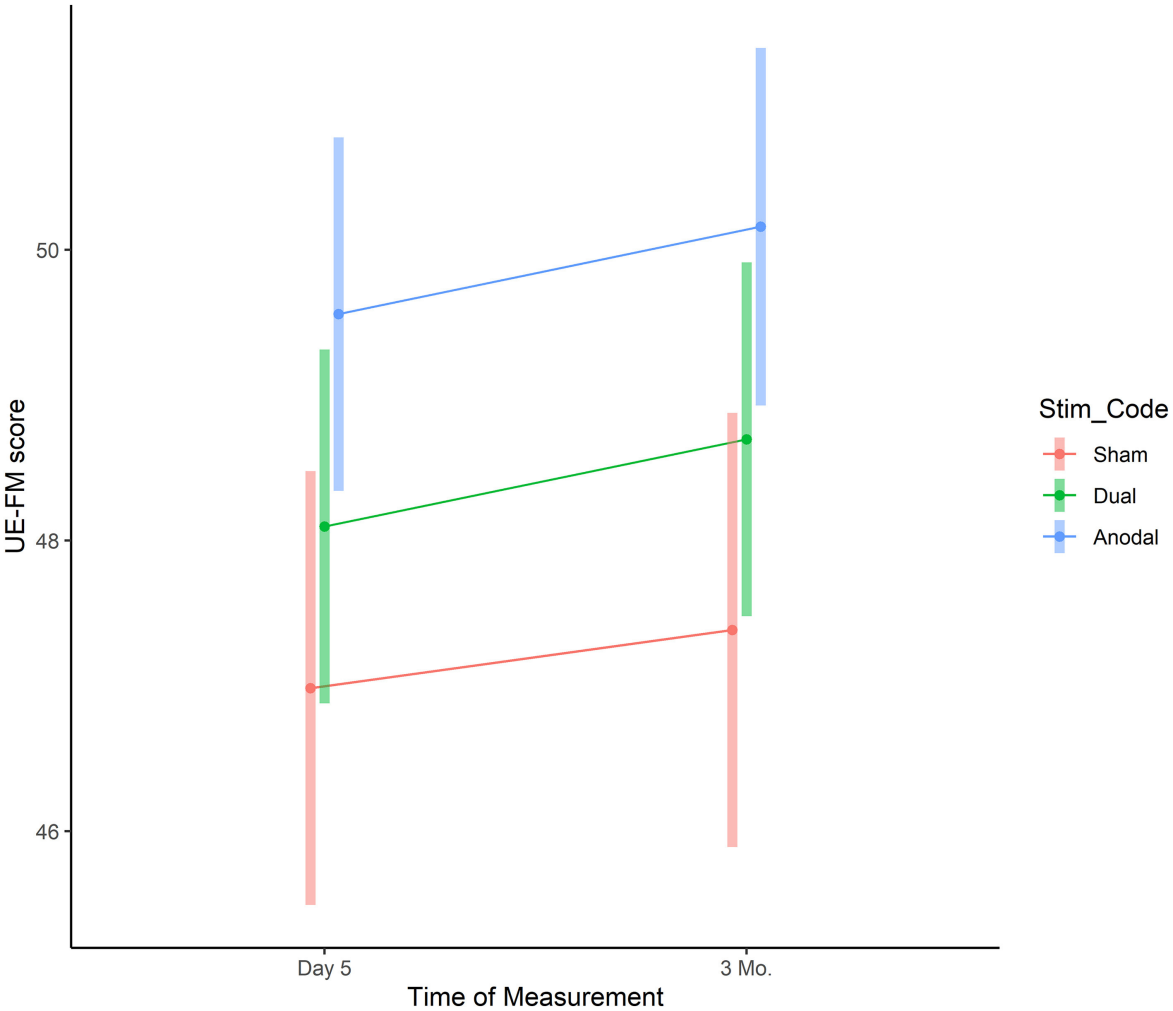
UE-FM assessment. The graphs depict the adjusted mean UE-FM score for each group at post-assessment and follow-up period based on the linear mixed model. Vertical bars represent model based 95% confidence intervals.

Adjusted UE-FM scores at day 5 were 47.0 (95%CI: 45.5–48.5) for sham, 48.1 (95%CI: 46.9–49.3, difference to sham: 1.1, 95%CI: −0.8 to 3.0, *p* = 0.251) for dual, and 49.6 (95%CI: 48.3–50.8, difference to sham: 2.6, 95%CI: 0.6–4.5, *p* = 0.010) for anodal (Figure 4). Three month later the adjusted UE-FM scores were: 47.4 (95%CI: 45.9–48.9) for sham, 48.7 (95%CI: 47.5–49.9, difference to sham: 1.3, 95%CI: −0.6 to 3.2, *p* = 0.177) for dual, and 50.2 (95%CI: 48.9–51.4, difference to sham: 2.8, 95%CI: 0.8–4.7, *p* = 0.006) for anodal. In the anodal stimulation group the values were highest. However, differences between anodal and dual were small at day 5 (mean difference: 1.5, 95%CI: −0.3 to 3.2, *p* = 0.094) and at 3 months follow up (mean difference: 1.5, 95%CI: −0.3 to 3.2, *p* = 0.095).

### Motor Training

**Figure 5:**
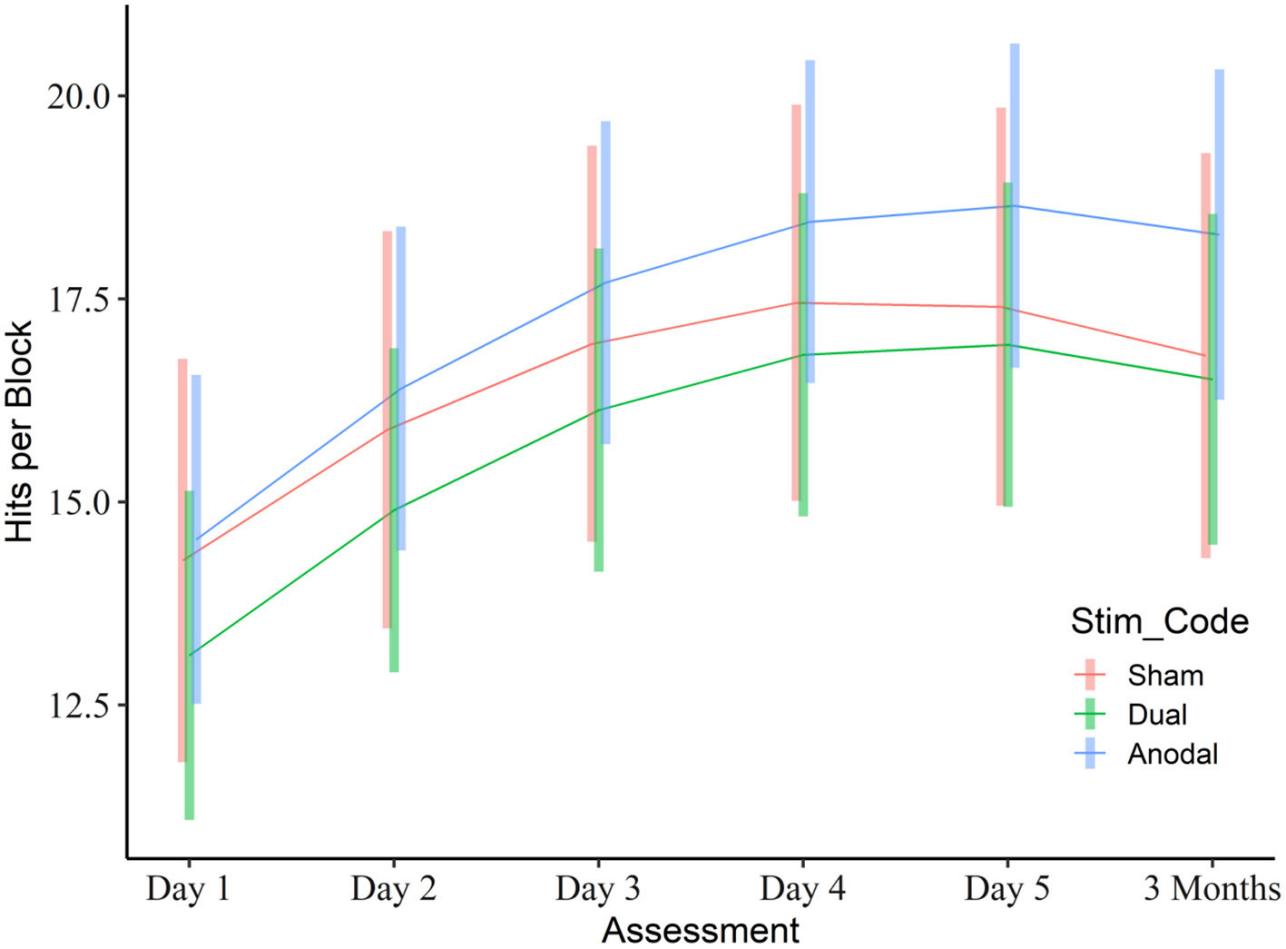
Learning curve in the visuo-motor grip force tracking task training task. The graph depicts the mean number of hits per block for each group across the training and follow-up period based on the linear mixed model. Vertical bars represent model based 95% confidence intervals.

There was no overall difference between the groups with regard to hits (reference sham: dual β = −0.73, 95%CI: −3.76 to 2.31, *p* = 0.64, *R*^2^ = 0.01; anodal β = 0.88, 95%CI: −2.16 to 3.91, *p* = 0.58, *R*^2^ = 0.01). There was a curvilinear improvement in performance (TIME^2^ β = −0.28, 95%CI: −0.34 to −0.21, *p* < 0.001, *R*^2^ = 0.03, TIME β = 0.50, 95%CI: 0.31–0.70, *p* < 0.001, *R*^2^ = 0.01), and this effect tended to be more pronounced over time in both active stimulation groups compared to sham (dual × time, β = 0.17, 95%CI: −0.08 to 0.43, *p* = 0.18, *R*^2^ = 0.00; anodal × time, β = 0.25, 95%CI: −0.007 to 0.50, *p* = 0.06, *R*^2^ = 0.00; Figure 5). Model based estimates at day 5 were 17.2 (95%CI: 14.7–19.6) for sham, 16.8 (95%CI: 14.8–18.8) for dual, and 18.5 (95%CI: 16.5–20.5) for anodal stimulation (pairwise comparisons: all *p* > 0.32).

### WMFT

**Figure 6:**
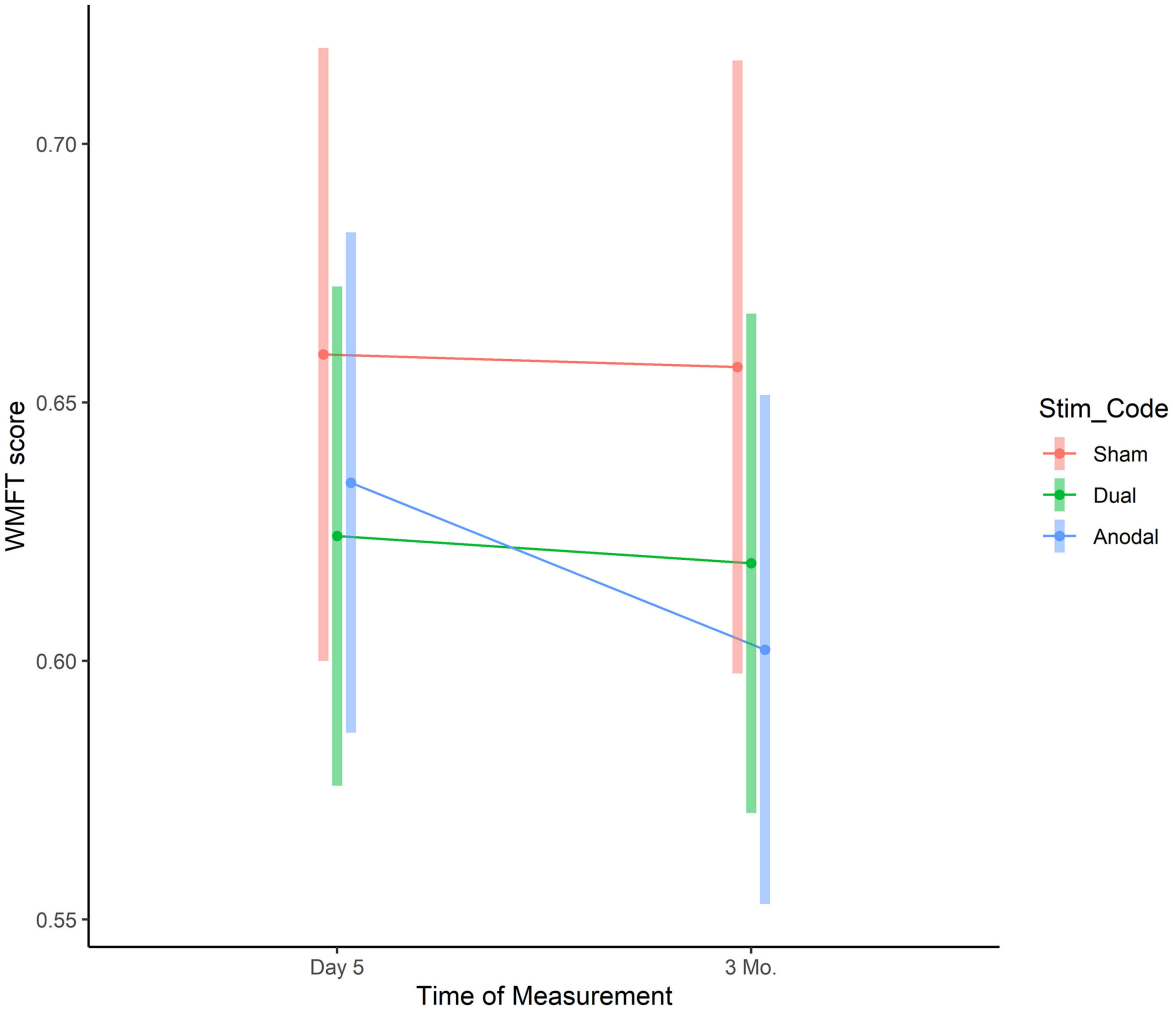
WMFT assessment. The graphs depict the mean WMFT completion time for each group across the training and follow-up period based on the linear mixed model. Vertical bars represent model based 95% confidence intervals.

Adjusted WMFT scores at day 5 were 0.66 (95%CI: 0.60–0.72) for sham, 0.62 (95%CI: 0.57–0.67, difference to sham: −0.04, 95%CI: −0.11 to 0.04, *p* = 0.360) for dual, and 0.63 (95%CI: 0.59–0.68, difference to sham: −0.02, 95%CI: −0.10 to 0.05, *p* = 0.518) for anodal (Figure 6). Three month later the adjusted WMFT scores were: 0.66 (95%CI: 0.60–0.72) for sham, 0.62 (95%CI: 0.57–0.67, difference to sham: −0.04, 95%CI: −0.11 to 0.04, *p* = 0.323) for dual, and 0.60 (95%CI: 0.55–0.65, difference to sham: −0.05, 95%CI: −0.13 to 0.02, *p* = 0.161) for anodal.

## Discussion

The results of this randomized, controlled clinical trial demonstrated that a visuo-motor grip force tracking task training, consisting of isometric abductions of the paretic thumb, improved control of the paretic thumb, and resulted in generalization to clinical assessments of upper extremity function such as UE-FM, but not WMFT, in chronic stroke patients. Compared to sham tDCS, UE-FM performance improvement was more pronounced in the group that had received unilateral (anodal) tDCS. However, this effect was relatively small (Cohen's *d* = 0.34) and those add-on effects were not significantly different from bilateral (dual) tDCS. No improvement on the WMFT was found.

Previous studies have reported that 4 weeks using comparable isometric pinch task resulted in better trained task performance and improvements of UE-FM, but not WMFT (38). Similarly, in a sample of well-recovered stroke patients, 1 week of training on a sequential visual isometric pinch force task resulted in better task performance, improvements in the Jebsen Taylor hand function test (JTT) and the Grooved Pegboard Test (GPT) (55). Our study is in line with these findings by showing that visuo-motor grip force tracking task training can result in long-term improvements of trained task performance and functionally relevant recovery of upper extremity function.

However, the present study did not confirm substantial add-on effects of unilateral or bilateral tDCS. While anodal tDCS selectively resulted in steeper gains on the UE-FU, these effects were small and not statistically different from bilateral stimulation. No stimulation effects were found on trained task performance or the WMFT.

Previous studies had only investigated effects of unilateral tDCS on visuo-motor grip force tracking task training and reported mixed results. While, Pavlova et al. (38) demonstrated beneficial effects of anodal tDCS on the shoulder-elbow subscale of the UE-FM, no effects on trained task performance were found. In contrast, Hamoudi et al. (55) reported improved trained task performance with anodal tDCS, but not on the JTT and GPT. Both studies reported no long-term effects of anodal tDCS on task performance, but beneficial effects on the shoulder-elbow subscale were maintained for up to 2 months (38). Therefore, mainly weak effects of anodal tDCS on visuo-motor grip force tracking task training have been reported previously. Together with the results of our own study, this questions the utility of tDCS to enhance the outcome of this particular type of training. Nonetheless, while there were no substantial effects at the group level, individual participants may have benefited from tDCS and future studies are need to include individualized modeling of current flow and functional imaging to investigate characteristics of potential responders.

### Limitations

The results of this study should be interpreted with caution due to the small sample size and high inter-individual variance in baseline motor function, lesion site, location and extent, time since stroke, age, and gender and missing data at the follow-up assessment (5/40 patients) and these factors may have contributed to the lack of effects in this study. Nonetheless, the missing data was balanced across the stimulation conditions and the mixed effects models used are robust regarding missing data. Furthermore, we also conducted a power analysis based on the strongest effect (i.e., the comparison of UE-FM scores between anodal vs. sham tDCS at day 5 and the 3 months follow up). At day 5, raw mean and SD values for anodal UE-FM were 50.4 (*SD*: 14.3), and for sham 44.6 (*SD*: 20.9) resulting in a standardized effect size (Cohen's d) of 0.34. To demonstrate effects of 0.34 or larger using a two sample *t*-test and a two-sided significance level of α = 0.05 and a power of 0.8, 139 individuals per group would have to be included for a significant effect in a new study. Please note, with the current sample size (*N* = 40), we achieved an effect size of 0.261 between the three groups (ratio of variance of the means by the within group variance; significance level of 0.05, power 80% in a one-way analysis of variance). This highlights that smaller proof-of-principle studies are imperative prior to investing limited resources into larger randomized controlled trials and our results in combination with previous studies do not encourage such follow-up trials for the combination of tDCS and visuo-motor grip force tracking task training.

### Conclusions and Outlook

Our results demonstrated significant performance improvements due to the visuo-motor grip force tracking task training in chronic patients with motor stroke. However, only limited add-on effects were induced by unilateral anodal tDCS. Dual tDCS did not improve training outcome.

## Data Availability Statement

The raw data supporting the conclusions of this article will be made available upon reasonable request.

## Ethics Statement

The studies involving human participants were reviewed and approved by Ethics Committee of the Charité Universitätsmedizin Berlin (Protocol: EA1/026/11). The patients/participants provided their written informed consent to participate in this study.

## Author Contributions

AF and RL conceived the study. BT, RD, DH, and JW processed the data. BT and UG performed the statistical analysis. BT wrote the manuscript. AF and MM revised the manuscript. All authors contributed to the article and approved the submitted version.

## Funding

This work was supported by the Else Kröner-Fresenius Stiftung (RL:2011-119), Deutsche Forschungsgemeinschaft (AF:DFG-Exc-257; SFB 1315, Project #327654276), and Bundesministerium für Bildung und Forschung (AF and MM:01EO0801).

## Conflict of Interest

The authors declare that the research was conducted in the absence of any commercial or financial relationships that could be construed as a potential conflict of interest.

## Publisher's Note

All claims expressed in this article are solely those of the authors and do not necessarily represent those of their affiliated organizations, or those of the publisher, the editors and the reviewers. Any product that may be evaluated in this article, or claim that may be made by its manufacturer, is not guaranteed or endorsed by the publisher.
