# Supplementary material for: Limited Add-On Effects of Unilateral and Bilateral Transcranial Direct Current Stimulation on Visuo-Motor Grip Force Tracking Task Training Outcome in Chronic Stroke. A Randomized Controlled Trial
Source: Front Neurol. 2021 Nov 11;12:736075. doi: 10.3389/fneur.2021.736075 (PMC8631774; doi:10.3389/fneur.2021.736075)
Supplement: Supplementary file 1 [file Data_Sheet_1.docx]

Supplementary Material

No add-on effects of unilateral and bilateral transcranial direct current stimulation on fine motor skill training outcome in chronic stroke. A randomized controlled trial

**Benedikt Taud^1^, Robert Lindenberg ^1,2,3^, Robert Darkow^1^, Jasmin Wevers^1^, Dorothee Höfflin^1^, Ulrike Grittnern^2,4^, Marcus Meinzer^1,2,5,†^, Agnes Flöel^1,2,5,6^**,**^†^.**

**† Contributed equally**

# Supplementary Analysis

UE-FM: Model based estimates at day 5 were 47.0 (95%CI: 45.5-48.5) for sham, and 48.8 (95%CI: 47.9-49.7) for active stimulation (mean difference: 1.8, 95%CI: 0.1-3.6, p=0.042), and at 3 months follow up: 47.4 (95%CI: 45.9-48.9) for sham, and 49.4 (95%CI: 48.5-50.3) for active stimulation (mean difference: 2.0, 95%CI: 0.3-3.8, p=0.025).

Motor task: There was overall no difference between the “active stimulation” and the sham group with regard to hits (reference sham: active β=0.07, p=0.958, R²<0.01). There was a curvilinear improvement in performance (TIME² β=-0.28, p<0.001, R²=0.03, TIME β=0.50, p<0.001, R²=0.01), but no substantial difference between active stimulation group and sham (active x time, β=0.21, p=0.069, R²=0.00). Model based estimates at day 5 were 17.2 (95%CI 14.7-19.6) for sham, and 17.7 (95%CI: 16.2-19.1) for active stimulation (p=0.727).

WMFT: Model based estimates at day 5 were 0.66 (95%CI: 0.60-0.72) for sham, and 0.63 (95%CI: 0.60-0.66) for active stimulation (mean difference: -0.03, 95%CI: -0.10-0.04, p=0.378), and at 3 months follow up: 0.66 (95%CI: 0.60-0.72) for sham, and 0.61 (95%CI: 0.58-0.65) for active stimulation (mean difference: -0.05, 95%CI: -0.11-0.02, p=0.180).

# Supplementary Tables

# S1. Estimates of linear mixed model motor function (UE-FM). (n=40 individuals, 79 measures) (random intercept model)

| **Fixed effects** |  | | | |
| --- | --- | --- | --- | --- |
|  | **beta** | **95%-CI** | **p** | ***Partial R²*** |
| Intercept | 2.4 | 0.1-4.7 | 0.049 |  |
| Baseline UE-FM Score | 1.0 | 0.9-1.0 | <0.001 | 0.99 |
| Stimulation  (ref: Sham) |  |  |  |  |
| Dual | 1.1 | -0.8-3.0 | 0.251 | <0.01 |
| Anodal | 2.6 | 0.7-4.4 | 0.010 | 0.03 |
|  |  |  |  |  |
| TIME point 3 month follow up (ref: day 5) | 0.4 | -0.5-1.3 | 0.388 | <0.01 |
| **Interaction** |  |  |  |  |
| Dual * TIME (3 mo) | 0.2 | -1.0-1.4 | 0.338 | <0.01 |
| Anodal * TIME (3 mo) | 0.2 | -1.0-1.4 | 0.337 | <0.01 |
| Random effects | sigma |  |  |  |
| subject | 2.1 | 1.6-2.7 |  |  |
| residual | 1.0 | 0.8-1.2 |  |  |
| Total R^2^ | 0.99 |  |  |  |

**S2. Model based marginal estimates, 95%CI and differences, UE-FM,** (n=40 individuals, 79 measures)

|  | **Day 5** | | **3 months follow up** | |
| --- | --- | --- | --- | --- |
|  | Adjusted mean, (95%CI) | Mean difference (95%CI) compared to sham, p | Adjusted mean, (95%CI) | Mean difference (95%CI) compared to sham, p |
| **sham** | 47.0 (45.5-48.5) |  | 47.4 (45.9-48.9) |  |
| **dual** | 48.1 (46.9-49.3) | 1.1 (-0.8-3.0) p=0.251 | 48.7 (47.5-49.9) | 1.3 (-0.6-3.2) p=0.177 |
| **anodal** | 49.6 (48.3-50.8) | 2.6 (0.6-4.5) p=0.010 | 50.2 (48.9-51.4) | 2.8 (0.8-4.7) p=0.006 |

**S3. Estimates of linear mixed model for the fine motor skill training. (n=40 individuals, 1864 measures) (random intercept model)**

| **Fixed effects** |  | | | |
| --- | --- | --- | --- | --- |
|  | **beta** | **95%-CI** | **p** | ***Partial R²*** |
| Intercept | 9.69 | 5.86-13.53 | <0.001 |  |
| Stimulation  (ref: Sham) |  |  |  |  |
| Dual | -0.73 | -3.76-2.31 | 0.641 | 0.01 |
| Anodal | 0.88 | -2.16-3.91 | 0.575 | 0.01 |
|  |  |  |  |  |
| Baseline UEFM | 0.13 | 0.06-0.20 | <0.001 | 0.27 |
| Training Blocks | 0.33 | 0.26-0.40 | <0.001 | 0.04 |
| TIME | 0.50 | 0.31-0.70 | <0.001 | 0.01 |
| TIME² | -0.28 | -0.34- -0.21 | <0.001 | 0.03 |
| **Interaction** |  |  |  |  |
| Dual * TIME | 0.17 | -0.08-0.43 | 0.179 | <0.01 |
| Anodal * TIME | 0.25 | -0.01-0.50 | 0.057 | <0.01 |
| Random effects | sigma |  |  |  |
| subject | 3.74 | 2.83-4.58 |  |  |
| residual | 3.60 | 3.49-3.72 |  |  |
| Total R^2^ | 0.36 |  |  |  |

**S4.Model based marginal estimates, 95%CI and differences, motor task, (n=40 individuals, 1864 measures)**

|  | **Day 5** | | **3 months follow up** | |
| --- | --- | --- | --- | --- |
|  | Adjusted mean, (95%CI) | Mean difference (95%CI) compared to sham, p | Adjusted mean, (95%CI) | Mean difference (95%CI) compared to sham, p |
| **sham** | 17.2 (14.7-19.6) |  | 16.3 (13.8-18.8) |  |
| **dual** | 16.8 (14.8.18.8) | -0.4 (-3.6-2.8) p=0.811 | 16.1 (14.0-18.2) | -0.2 (-3.4-3.0) p=0.899 |
| **anodal** | 18.5 (16.5-20.5) | 1.4 (-1.8-4.5) p=0.390 | 17.9 (15.8-20.0) | 1.6 (-1.6-4.8) p=0.319 |

**S5. Estimates of linear mixed model motor impairment (WMFT). (n=40 individuals, 79 measures) (random intercept model)**

| **Fixed effects** |  | | | |
| --- | --- | --- | --- | --- |
|  | **beta** | **95%-CI** | **p** | ***partialR²*** |
| Intercept | 0.01 | -0.06-0.08 | 0.767 |  |
| Baseline WMFT | 0.95 |  | <0.001 | 0.98 |
| Stimulation  (ref: Sham) |  |  |  |  |
| Dual | -0.04 | -0.11-0.04 | 0.360 | 0.02 |
| Anodal | -0.02 | -0.10-0.05 | 0.518 | 0.01 |
| TIME point 3 month follow up (ref: day 5) | -0.002 | -0.05-0.04 | 0.917 | <0.01 |
| **Interaction** |  |  |  |  |
| Dual * TIME (3 mo) | -0.003 | -0.06-0.06 | 0.926 | <0.01 |
| Anodal * TIME (3 mo) | -0.03 | -0.09-0.03 | 0.335 | 0.01 |
| Random effects | sigma |  |  |  |
| subject | 0.60 | 0.48-0.76 |  |  |
| residual | 0.06 | 0.05-0.07 |  |  |
| Total R^2^ | 0.98 |  |  |  |

**S6. Model based marginal estimates, 95%CI and differences, WMFT,** (n=40 individuals, 79 measures)

|  | **Day 5** | | **3 months follow up** | |
| --- | --- | --- | --- | --- |
|  | Adjusted mean, (95%CI) | Mean difference (95%CI) compared to sham, p | Adjusted mean, (95%CI) | Mean difference (95%CI) compared to sham, p |
| **sham** | 0.66 (0.60-0.72) |  | 0.66 (0.60-0.72) |  |
| **dual** | 0.62 (0.58-0.67) | -0.04 (-0.11-0.04) p=0.360 | 0.62 (0.57-0.67) | -0.04 (-0.11-0.04) p=0.323 |
| **anodal** | 0.63 (0.59-0.68) | -0.02 (-0.10-0.05) p=0.518 | 0.60 (0.55-0.65) | -0.05 (-0.13-0.02) p=0.161 |

**Supplementary Figures**


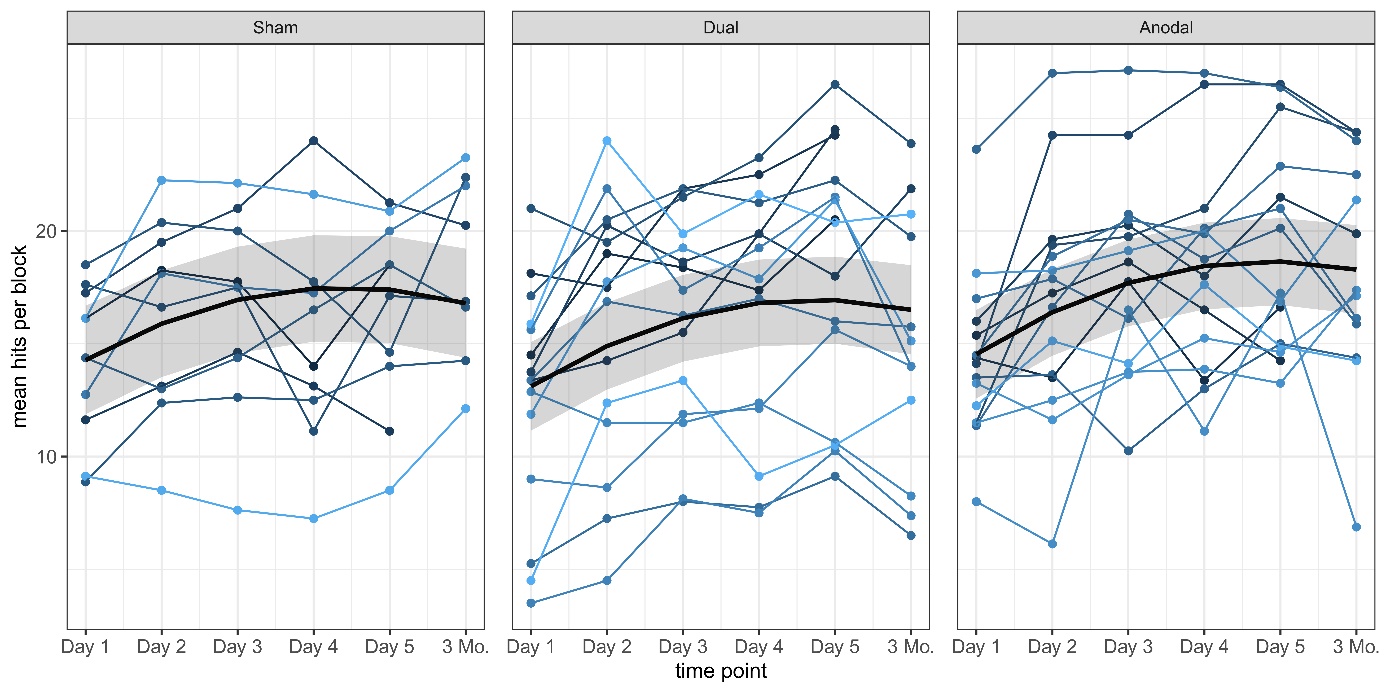
**Supplementary Figure 1**: Scatterplot of individual mean hits during the viuo-motor grip force tracking task per block and by time point of assessment and stimulation group, regression lines and 95%CI

*
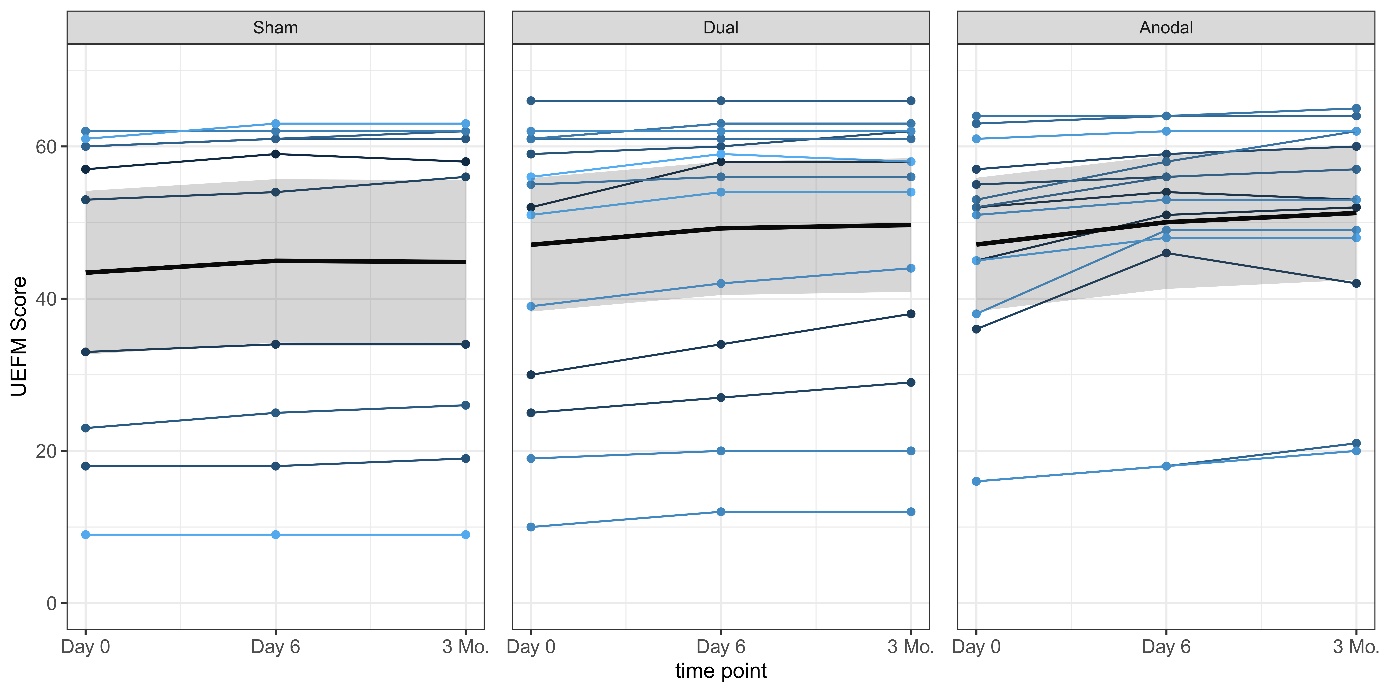
*

**Supplementary Figure 2:** Scatterplot of individual UE-FM scores by time point of assessment and stimulation group, regression lines and 95%CI

***
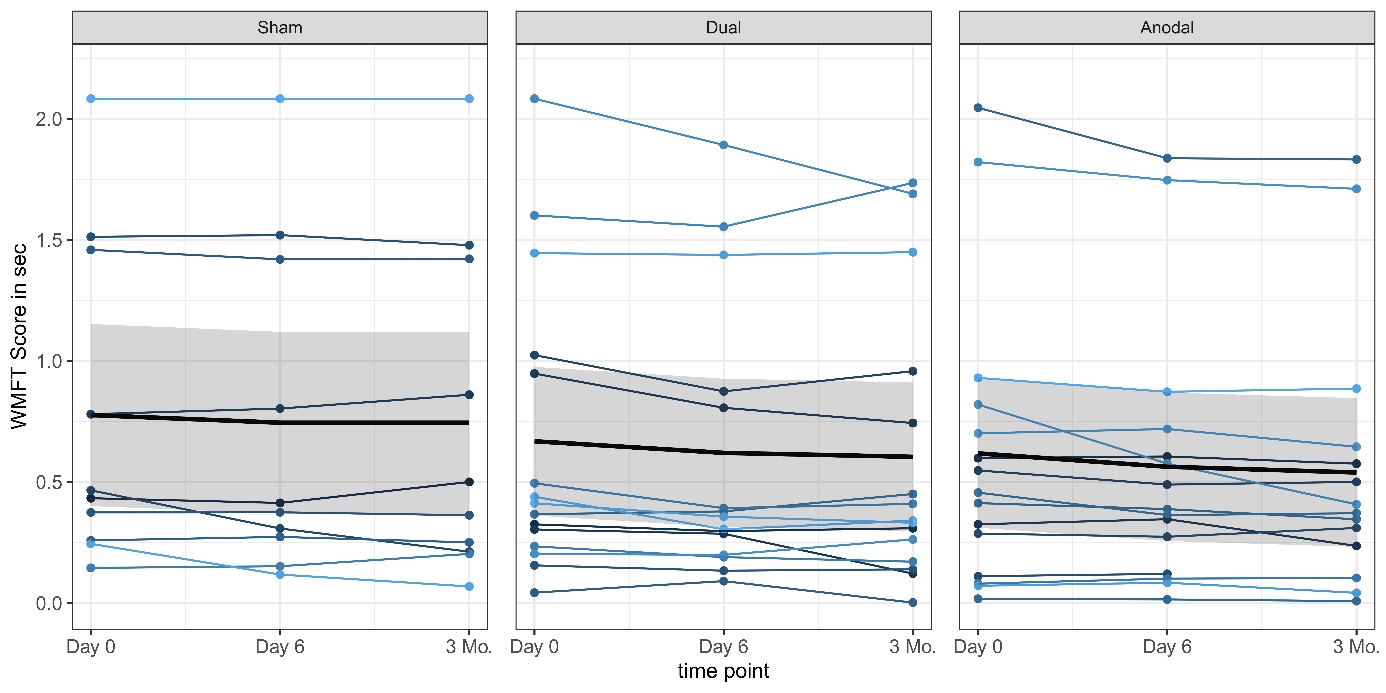
***

**Supplementary Figure 3**: Scatterplot of individual WMFT scores by time point of assessment and stimulation group, regression lines and 95%CI
